# Polyurethane Foam Chemical Recycling: Fast Acidolysis with Maleic Acid and Full Recovery of Polyol

**DOI:** 10.1021/acssuschemeng.3c07040

**Published:** 2024-03-07

**Authors:** Baoyuan Liu, Zach Westman, Kelsey Richardson, Dingyuan Lim, Alan L. Stottlemyer, Thomas Farmer, Paul Gillis, Nasim Hooshyar, Vojtech Vlcek, Phillip Christopher, Mahdi M. Abu-Omar

**Affiliations:** † Department of Chemistry and Biochemistry, University of California, Santa Barbara, California 93106, United States; ‡ Department of Chemical Engineering, University of California, Santa Barbara, California 93106, United States; § The Dow Chemical Company, Midland, Michigan 48640, United States; ∥ The Dow Chemical Company, Herbert H Dowweg 5, Hoek 4542 NH,The Netherlands

**Keywords:** polyurethane, acidolysis, recycled polyol, maleic acid

## Abstract

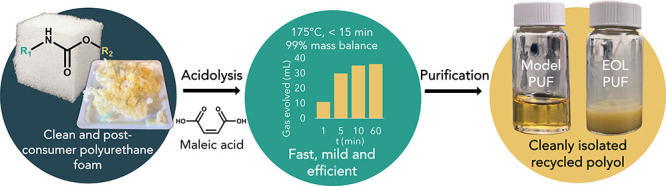

Chemical recycling of polyurethane (PU) waste is essential to displace the need for virgin polyol production and enable sustainable PU production. Currently, less than 20% of PU waste is downcycled through rebinding to lower value products than the original PU. Chemical recycling of PU waste often requires significant input of materials like solvents and slow reaction rates. Here, we report the fast (<10 min) and solvent-free acidolysis of a model toluene diisocyanate (TDI)-based flexible polyurethane foam (PUF) at <200 °C using maleic acid (MA) with a recovery of recycled polyol (repolyol) in 95% isolated yield. After workup (hydrolysis of repolyl ester and separations), the repolyol exhibits favorable physical properties that are comparable to the virgin polyol; these include 54.1 mg KOH/g OH number and 624 cSt viscosity. Overall, 80% by weight of the input PUF is isolated into two clean-cut fractions containing the repolyol and toluene diamine (TDA). Finally, end-of-life (EOL) mattress PUF waste is recycled successfully with high recovery of repolyol using MA acidolysis. The solvent-free and fast acidolysis with MA demonstrated in this work with both model and EOL PUF provides a potential pathway for sustainable and closed-loop PU production.

## Abstract 2

Implementing PUF recycling with neat dicarboxylic acid without solvent to recover the major component polyol fosters a circular economy by reducing waste and promoting sustainable production of PUF.

## Introduction

PU (polyurethane) is synthesized from the reaction of polyol and diisocyanate. PU is the sixth most produced polymer and is used in a variety of products, ranging from clothing and furniture to adhesives, insulations, and automobiles.^1−4^ The global market size of PU was 37.8 billion USD in 2020 and is expected to grow to a predicted market size of 88.8 billion USD by 2030.^5,6^ The increasing demand for PU products raises concerns about its sustainable production and the fate of PU waste.

Currently, the production of PU depends on nonrenewable carbon resources.^7^ The 2900 kilo metric tons (kt) of PU produced in the United States in 2016 consumed 1100 kt of crude oil and 1100 kt of natural gas.^8^ Renewable feedstock alternatives, such as polyols made from vegetable oil and biomass, have been investigated for PU synthesis.^7,9−12^ While biobased PU has a more renewable feedstock and may be more biodegradable than conventional PU, it is often limited in its functional properties. Furthermore, the high cost of biobased polyols has limited commercialization of biobased PU.^13^

Despite the push toward sustainable virgin PU synthesis, improvement of recycling processes for PU waste has remained underdeveloped. In the United States in 2016, 2000 kt of PU waste was discarded, while only 390 kt was recycled and returned to the market as lower value products.^8^ The only PU recycling method available at a commercial scale in the United States is mechanical rebinding, which combines shredded PU waste with binders through a continuous molding process to make new PU products. However, PU materials are thermoset plastics with highly cross-linked structures, so it is difficult to mechanically recycle PU waste.^14^ As a result, products from the mechanical recycling of PU are usually low value products, such as carpet underlayment.^8^ It is therefore of interest to develop methods to recycle and isolate the chemical components of PU, which could be used directly in making new PU materials (closed loop recycling).

Chemical recycling of PU involves the use of chemical reagents and heat to cleave urethane bonds and produce recycled polyol (repolyol), offering an opportunity to make new PU products with a higher value than can be achieved through mechanical methods.^3,15−17^ Recycling the polyol component of PU could have a significant impact on the sustainability of PU production, as 60% of primary energy use in PU manufacturing is associated with polyol production.^8^ Chemical processes such as hydrolysis, acidolysis, glycolysis, or aminolysis have shown promise for recovering repolyol from PU materials; however, they have drawbacks that have thus far prevented their widespread use. Aminolysis uses amines, which impose safety and environmental concerns.^18−22^ Hydrolysis requires a high energy input and long reaction time.^23−27^ Glycolysis has been well studied and developed to a pilot scale (Lymtal International Inc.). However, it requires a 2–5 times glycol reagent input per PU mass.^19,28−30^ The advancement of the PU chemical recycling process, in terms of commercialization, is currently in its early stage of development. Notably, key players in the global plastics market, including Evonik, REMONDIS, Covestro, and Dow, have introduced sustainable solutions to the EU market that integrate PU production and recycling. These initiatives are branded PUReSmart (Covestro) and Renuva (Dow).

Acidolysis, the reaction of PU with organic acids, is another promising method for PU waste valorization. Recent studies by Gama et al. and others have shown that dicarboxylic acids (DCAs), such as succinic, phthalic, and adipic acid, can decompose flexible PU foams (PUF) into repolyol and amides.^17,31,32^ While acidolysis shows good material and energy efficiency and low toxicity compared to other methods, PU decomposition with DCAs remains underdeveloped. Previous literature has reported long reaction times (3–6 h) at relevant reaction conditions (<200 °C to avoid PU thermal decomposition), suggesting slow rates of reaction. Furthermore, there is a lack of detailed, quantitative analysis for separations and treatment procedures to recover the repolyol and amide products in high yields.

Here, we describe a PUF acidolysis reaction with maleic acid (MA) that reaches completion in <15 min at 175 °C under 1 atm nitrogen (N_2_) in neat conditions without using a solvent, a notable improvement on previous methods reported in the literature. At the end of the reaction, marked by stoichiometric CO_2_ evolution, the PUF is fully degraded into repolyol ester and amide products with a 98% mass balance closure. Hydrolysis followed by a separation process is used to isolate repolyol with physical properties comparable to those of virgin polyol. Thus, a quantitative and near-stoichiometric repolyol yield is achieved via acidolysis, as well as valorization of the amide coproduct to TDA (toluene diamine), a PUF precursor.^27^ Characterization of the acidolysis reaction of PUF with MA and its product streams along with a repolyol purification strategy are detailed. The application of this chemical recycling methodology is further demonstrated on real end-of-life (EOL) mattress PUF mixed waste, highlighting the robust nature of the chemistry.

## Results

In this work, both the model and EOL PUF were used as starting materials to demonstrate PUF chemical recycling through acidolysis. The model foam was composed of TDI (toluene diisocyanate) and VORANOL 8136, a polyether-based polyol, both of which are common reagents for the construction of flexible polyurethane foams; therefore, the model foam is expected to be representative of a typical flexible PUF. The EOL PUF was collected from discarded European mattresses without further purification. MA was selected for acidolysis because it is a linear organic diacid with little steric hindrance, has high commercial availability via its anhydride, has a low melting point of 136 °C, and is strongly acidic due to intramolecular hydrogen bonding (p*K*_a__1_ = 1.90, p*K*_a__2_ = 6.07, Table S1).

**Figure 1:**
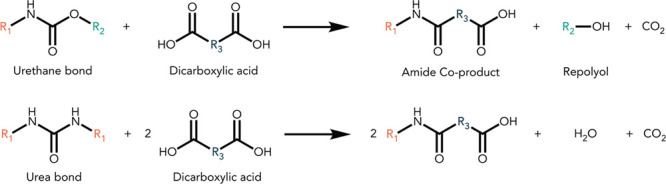
Reaction scheme for acidolysis with maleic acid (MA) of (a) urethane bonds and (b) urea bonds in polyurethane foam (PUF).

**Figure 2:**
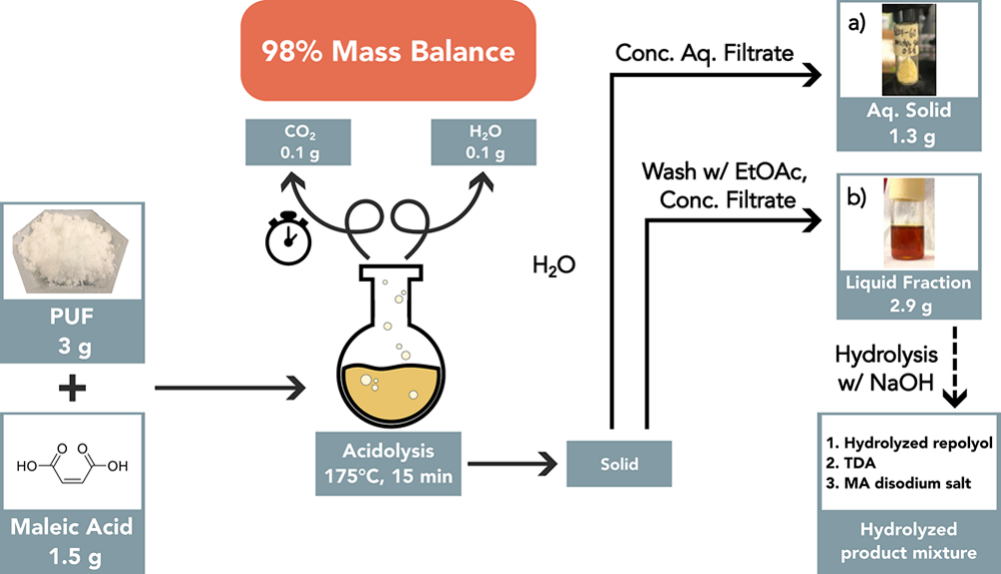
Reaction workup and mass balance of a typical acidolysis of model PUF with MA at a PUF/MA 1:0.5 ratio. Acidolysis conditions: 175 °C for 15 min. The cooled post reaction mixture was washed with water and EtOAc followed by liquid–liquid phase separation via centrifuge. The fraction (a) was the dried solute obtained from aqueous phase, while the fraction (b) was the remaining component obtained from organic phase after removal of EtOAc.

The starting slabs of PUF were shredded into finer particles. According to SEM (scanning electron microscope) analysis, the model PUF shreds (white powder) were between 500 and 2000 μm (Figure S1b), while the EOL PUF particles (yellow powder) were in the range of 155–750 μm (Figure S1d). Compared to the intact foam (Figure S1a,c), it is apparent that many of the struts and cells on the PUF polymer network were destroyed by the shredding process. However, the FT-IR spectra of PUF samples before and after grinding were nearly identical (Figure S2a,b). This implies that mechanical grinding induced only physical structural changes and not chemical degradation. PUF decomposition (Figure 1) through acidolysis of the urethane and urea linkages to repolyol, amide, and CO_2_ was induced only by the reaction between PUF and MA. Figure 2 illustrates the reaction and separation process and mass balance of a typical acidolysis reaction with 3.0 g of PUF and 1.5 g of MA. The reaction temperature was 175 °C to avoid thermal decomposition of the PUF (i.e., the reversible thermal cleavage of urethane bonds to form polyol and isocyanate and/or urea bonds to form isocyanate and water), which was determined by TGA (thermogravimetric analysis) to start between 200 and 220 °C for both the model and EOL PUF (Figure S3). After a 15 min reaction at 175 °C, a 98% mass balance was achieved. The solute collected in the aqueous phase (Figure 2a) was determined by NMR to be unreacted excess MA. The dried product mixture was a viscous brown liquid (Figure 2b). The repolyol and amide products from PUF acidolysis were extracted from the viscous liquid by using ethyl acetate (EtOAc). Evaporated water from the reaction (0.1 g) was condensed in a cold finger and weighed; 0.1 g CO_2_ was produced during acidolysis and quantified by purging the resulting gas from the reaction into a saturated Ca(OH)_2_ solution and isolating CaCO_3_. Additionally, a solid residue was observed at a longer reaction time (i.e., 1–3 h), which was determined by FT-IR to be fumaric acid (Figures S4 and S5b), resulting from the isomerization of MA.

**Figure 3:**
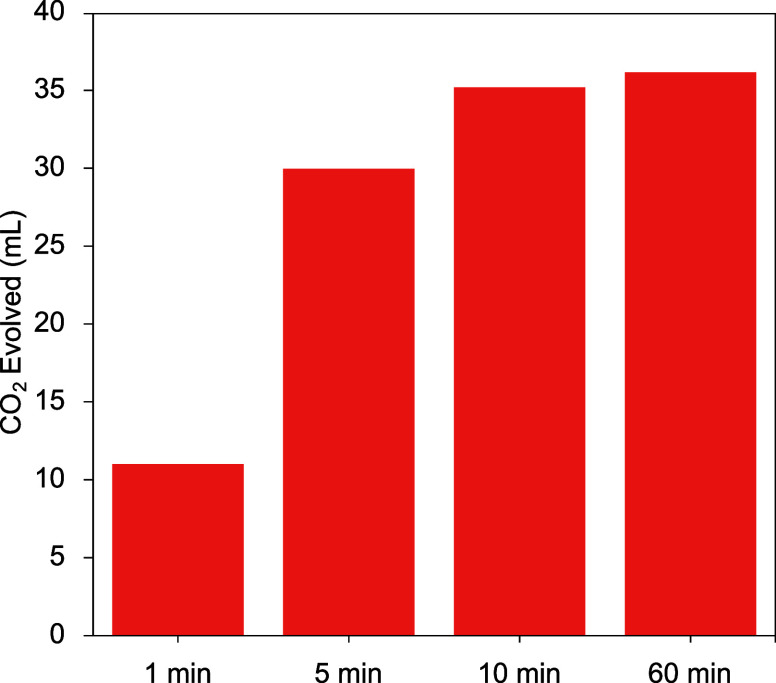
Gas (CO_2_) generation observed in real time for the acidolysis of model PUF with MA. Reaction conditions: PUF = 1 g, MA = 3 g, 175 °C, 10 min.

Notably, according to the reaction scheme (Figure 1a,b), the CO_2_ generation was proportional to the complete decomposition of urethane and urea bonds, which were separately quantified. As a result, the degree of PUF decomposition could be monitored by quantifying the volume of CO_2_ from the acidolysis reaction. A gas evolution buret was connected to the acidolysis reaction to measure CO_2_ generation during PUF acidolysis (Figure S6). Figure 3 shows that during the first minute of reaction (time = 0 was denoted as when the flask reached 175 °C), 11 mL of gas was collected in the buret. The evolution of gas increased, reaching a plateau at ∼35 mL in ca. 10 min. This suggests that PUF acidolysis reached completion within ca. 10 min at 175 °C. Moreover, there was no solid residue left in the reaction vessel after 15 min of reaction.

**Figure 4:**
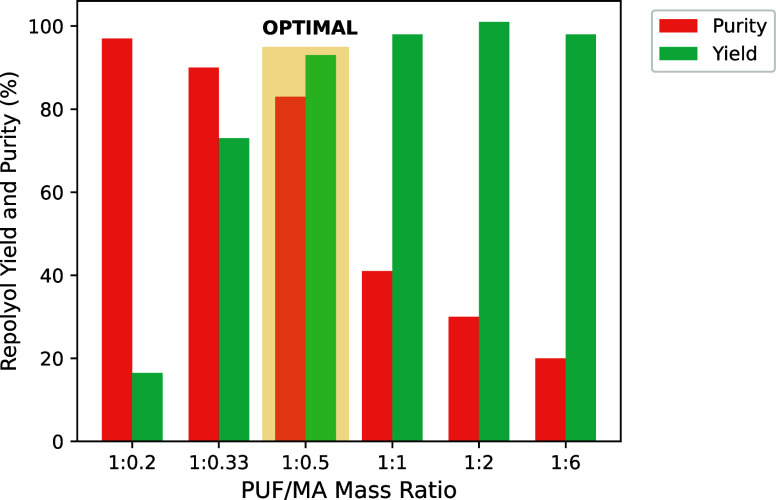
Repolyol yield (green) and purity (orange) in recovered liquid fraction (Figure 2b) after acidolysis of model PUF with MA. Reaction conditions: 175 °C, 15 min.

The analysis of liquid products was carried out by ^13^C NMR. Three major components were observed: unreacted maleic acid (δ^13^C 130.5 and 167.2 ppm), amides (δ^13^C 110–140 and 160–174 ppm), and repolyol (δ^13^C 16–20 and 62–80 ppm). Comparing the content of polyol within the model PUF to the amount of repolyol by quantitative ^13^C NMR, the yield of repolyol was determined. Figure 4 shows the yield (green charts) of repolyol obtained in the liquid fraction at different PUF/MA loading ratios (w/w). The minimum amount of MA to drive the PUF decomposition to near completion in 15 min was between 1:0.5 and 1:1 PUF:MA, where the yield of repolyol reached 93–98%. However, the purity (Figure 4, orange chart) of the repolyol, defined as the ratio of the mass of repolyol to total mass of the liquid fraction (Figure 2b), decreased from 80% at a 1:0.5 of PUF/MA ratio to 40% at 1:1 PUF/MA. The reduced purity of repolyol was due to the increased MA content in the liquid fraction, as a result of a large excess of unreacted MA. Therefore, the optimal PUF/MA loading was determined to be 1:0.5 in the 15 min reaction, which maintained a high yield and high purity of repolyol in the liquid product mixture (Figure 2b), as a result of not using excessive MA input.

**Figure 5:**
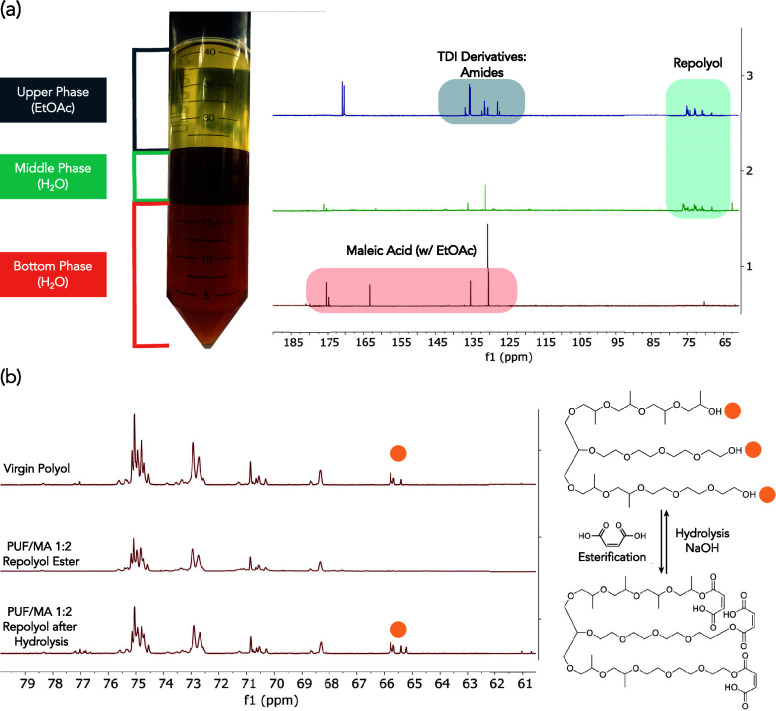
(a) Separation of the liquid fraction product from PUF acidolysis shown alongside the corresponding ^13^C NMR spectra for each layer; (b) ^13^C NMR spectra of the repolyol product before and after hydrolysis compared to virgin polyol (VORANOL 8136).

To extract the repolyol products out of the liquid fraction, a separation strategy was developed using EtOAc and aqueous NaOH (see the Experimental Section for details). After centrifugation, a three-phase separation of the liquid fraction was obtained (Figure 5a). According to ^13^C NMR analyses, the top EtOAc layer contained mainly amide products, with a small amount of repolyol. The majority of the repolyol product was isolated in the middle phase without apparent contaminants, except EtOAc (^13^C spectra in Figure 5a). The bottom aqueous layer contained mostly leftover excess MA with some residual EtOAc. With 2–3 repeats of this separation treatment, a high-purity repolyol product was obtained from the middle phase accounting for 92% isolated yield based on the polyol content in the original model PUF substrate. Notably, when the PUF/MA ratio in the acidolysis reaction was above 1:1, additional repeats of the separation treatment were required to obtain the purified repolyol, resulting in a lower repolyol isolated yield, 78% isolated repolyol from a PUF/MA ratio of 1:3 based on mass.

The ^13^C NMR spectrum (middle panel of Figure 5b) of the isolated repolyol is almost identical to a sample of the virgin polyol (VORANOL 8136), top panel of Figure 5b; however, the chemical shifts between 65 and 66.7 ppm, assigned to terminal −OH groups of the polyol, were absent from the isolated repolyol. This suggested that the repolyol must have reacted further with excess MA to form an ester. Indeed, analysis of the isolated repolyol by APC (aquagel porous chromatography) GPC (gel permeation chromatography) afforded a number-average molecular weight (*M*_n_) of 3213 g mol^–1^ (Figure S7), compared to the *M*_n_ of 2891 g mol^–1^ for VORANOL 8136. The difference in mass corresponds to the repolyols’ terminal −OH groups reacting to make polyol ester. To restore the −OH on the repolyol, a hydrolysis reaction was performed with NaOH(aq) (Figure 5b). After a 30 min hydrolysis reaction, δ^13^C signals between 65 and 66.7 ppm were observed for the hydrolyzed repolyol (bottom panel of Figure 5b). As a result, a purified repolyol with spectroscopic features (^13^C NMR and GPC) identical to those of the original polyol (VORANOL 8136) was isolated from our fast PUF acidolysis reaction with high yields. Additionally, the MA incorporated into the polyol-ester underwent neutralization as MA salt after hydrolysis workup (Figure 2b), and it was washed off during the purification of repolyol.

To simplify repolyol separation from the acidolysis product mixture, a two-step hydrolysis workup and purification treatment was implemented without the need to preseparate the repolyol from the liquid fraction. Although all the MA loaded for acidolysis was neutralized to MA salt at the end, the improved method not only reduces the total material (solvent) input required to process the product mixture but also offers simultaneous hydrolysis of amide products to form TDA, a precursor to TDI. After the 15 min PUF acidolysis reaction, a 25 mL aqueous NaOH solution was added directly to the acidolysis product mixture and held for 30 min at 150 °C. Subsequently, liquid–liquid extraction was performed with toluene to afford clean-cut fractions of hydrolyzed repolyol (see the Experimental Section for details). After phase separation from toluene, TDA was maintained in the aqueous phase, which was then isolated by EtOAc and characterized by GC-MS (gas chromatography-mass spectrometry) and 2D ^1^H–^15^N HSQC (heteronuclear single quantum coherence) NMR (Figures S8 and S9). The isolation yields of TDA and repolyol were 60–65% and 93–95%, based on their concentrations in the starting PUF substrate, which corresponded to 12–13 and 66–68 wt % of the input model PUF, respectively. Eventually, MA disodium salts formed during the reaction workup, and a small fraction of residual organic components were left in the aqueous phase.

There are several standard physical properties used to evaluate polyol/repolyols. These include hydroxyl number (OH_number_), viscosity, amine number, acid value (AV), water content, and molecular weight, which can be used to examine functionality of the polyol and gauge its suitability for the production of new PU materials.^33−36^ Accordingly, the repolyol obtained in this work was analyzed and compared with the virgin polyol VORANOL 8136. Table 1 summarizes the key properties of the isolated repolyol as well as virgin polyol. The OH_number_ of 54.1 mg of KOH/g obtained for the isolated repolyol falls in the middle of the range for VORANOL 8136 (52.8–56.1 mg of KOH/g). The viscosity of 624 cSt is within the range of what is considered ideal (550–650 cSt) to produce PUF. However, our isolated repolyol showed a significant amine number (1.633 mg KOH/g), AV (6.139 mg KOH/g), and water content (0.262 wt %) when compared to the virgin polyol, which may affect its processing to create new foams from the repolyol. Virgin polyol prepared from ethylene and propylene oxide contains no amines and has a very low water content (0.08 wt %). The molecular weights (*M*_n_) of repolyol and virgin polyol esters as determined by APC GPC in THF (tetrahydrofuran) were essentially identical (Figure S7, *M*_n_ = 3.21 and 3.39 kg/mol, respectively). This analysis suggests the repolyol obtained through the approach demonstrated here has excellent potential for closed-loop PUF production.^37^

**Table 1:** Key Physical Properties of Repolyol

characterization	analysis method	repolyol	VORANOL 8136
OH number (mg KOH/g)	ASTM D4274	54.1	52.8–56.1
Viscosity (cSt)	D4878	624	550–650
Amine number (mg KOH/g)	D6979	1.633	N/A
Acid value (mg KOH/g)	D7253	6.139	N/A
Water content (wt %)	E203	0.262	0.08
*M*_n_ (kg/mol)	GPC	3.21	2.89
Polydispersity	GPC	1.25	1.17

**Figure 6:**
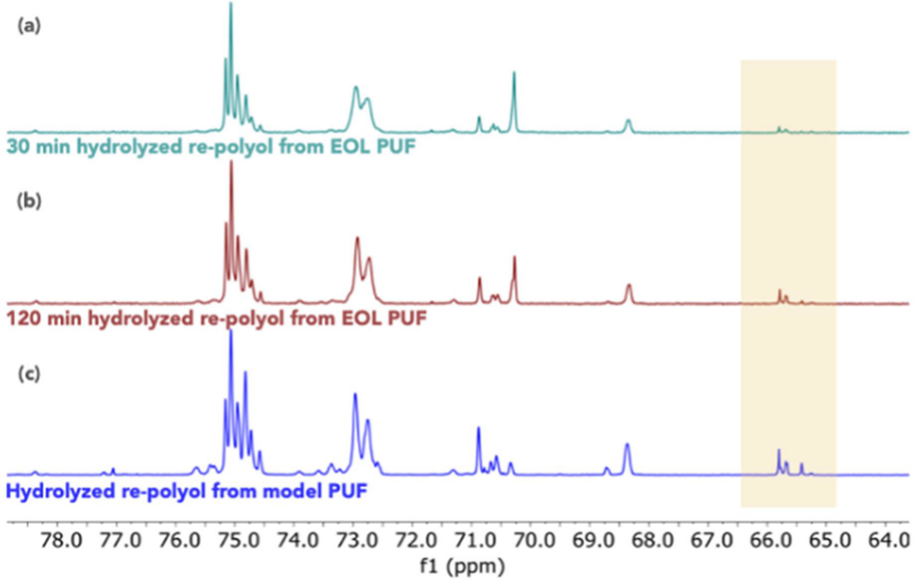
^13^C NMR spectra of (a) 30 min hydrolyzed EOL PUF repolyol; (b) 120 min hydrolyzed EOL repolyol; (c) 30 min hydrolyzed model PUF repolyol. Highlighted regions represent −OH end groups.

To validate the applicability of our PUF acidolysis with MA, subsequent hydrolysis, and separation methods, EOL PUF collected from European mattress waste was used as a substrate. The shredded EOL PUF was loaded with a 1:1 ratio (w/w) of MA. After holding the temperature at 175 °C for 15 min, a homogeneous liquid mixture was observed. This observation implied that the EOL PUF was fully decomposed under the same acidolysis conditions and with a comparable reaction rate to the model PUF. The obtained EOL repolyol was hydrolyzed and isolated as described in the two-step hydrolysis method above. Figure 6 compares the ^13^C NMR spectra of hydrolyzed repolyol from model PUF (Figure 6c) to those of EOL repolyol after 30 min hydrolysis (Figure 6a) versus 120 min hydrolysis (Figure 6b). Even though the virgin polyol(s) used in making the mattress waste is unknown and most likely a complex mixture of polyols, rather than the singular VORANOL 8136 polyol used in our model PUF, the ^13^C NMR spectrum of the repolyol from EOL PUF showed signals similar to those of VORANOL 8136, indicating the general features of a polyether polyol. Notably, the EOL repolyol obtained from a 120 min hydrolysis reaction showed greater quantities of −OH end group (δ^13^C 65–66.7) than from a 30 min hydrolysis, indicating the need for a longer hydrolysis reaction time for the EOL PUF than for the model PUF. Nevertheless, hydrolysis for 120 min yielded a clean product of repolyol, showing that fast PUF acidolysis is viable for recycling commercial EOL PUF waste into repolyol.

Previous literature has reported incomplete acidolysis reactions above 190 °C^32^^,^^33^ whereas we observe quantitative polyol release at 175 °C, suggesting a complete reaction. To confirm that complete polyol release is reasonable, DFT (density functional theory) calculations of the reaction-free energy were performed using a simplified urethane molecule at the reaction temperatures (see Supporting Information). These calculations show that the reaction is strongly exothermic, illustrating that a complete reaction under our conditions is reasonable and consistent with reaction thermodynamics.

## Discussion

We have demonstrated an effective PUF chemical recycling process, including a grinding pretreatment, fast and solvent-free acidolysis, hydrolysis to recover the −OH end groups on the recycled polyol, and product isolation. The grinding pretreatment facilitates rapid wetting of the PUF by liquefied MA, allowing fast reaction times of ca. 15 min at 175 °C. In contrast, the use of nongrinded PUF chunks (1–2 cm) as a feedstock at 3 g scale required >1 h to complete the acidolysis reaction that occurred in <15 min with the ground PUF. Because PUF is a low-density material, the grinding pretreatment also decreases the required reactor volume and improves heat transfer.

TGA analysis of the model PUF (Figure S3) showed that thermal degradation of the foam begins at 210–220 °C. A two-stage weight loss was observed. Comparing the percentage of weight loss for each stage to the composition of the model PUF (polyol and TDI), the first stage weight loss (25% by weight) is attributed to the thermal decomposition of PUF hard segments (oligomers of aromatic urea linkages = reaction product of TDI and water), while the second stage weight loss (72% by weight) corresponds to the decomposition of PUF soft segments. Since all reactions run well below the thermal degradation temperature of the PUF, the observed products and reactions are due to acidolysis and not thermal decomposition. This was confirmed by an isothermal TGA analysis of the PUF at 195 °C, which showed no weight loss over 15 min.

While mechanistic details remain to be investigated, MA induces cleavage of urethane and urea bonds, allowing the release of CO_2_, polyol, and aromatic amide components of the PUF. The cis-confirmation of MA may facilitate hydrogen bonding and thus proton transfer to urethane and urea bonds. The isomerization of MA to the trans isomer, fumaric acid (p*K*_a__1_ = 3.03, p*K*_a__2_ = 4.44), was observed at longer reaction times. For example, PUF acidolysis at 175 °C for 3 h produced a significant amount of fumaric acid (Figure S5b). However, mixing fumaric acid and PUF at 175 °C resulted in no decomposition of the polymer over 3 h. The inability of fumaric acid to react with PUF is because its high melting point of 286 °C prevents effective contact with PUF at the reaction temperature.^31^ It should be noted that the isomerization of MA to fumaric acid is slower than PUF acidolysis and can thus be entirely avoided in shorter reaction times. For instance, no fumaric acid was detected from a 15 min acidolysis reaction at 175 °C.

The crude liquid product mixture obtained from PUF acidolysis (Figure 2b) was composed of unreacted MA, amide, and repolyol. The degree of PUF decomposition at the lowest tested PUF/MA ratio of 1:0.2 (w/w) was low, with a yield of repolyol of less than 20% (Figure 4 green chart). It is worth noting that even at the lowest PUF/MA ratio, the moles of −COOH are still in excess of the total moles of urethane and urea linkages (∼1.7:1 mol-COOH/mol(urethane + urea)). After holding the reaction for more than 3 h at 175 °C for the PUF/MA 1:0.2 (w/w) ratio, acidolysis did not reach completion. This may in part be due to the inability of both −COOH groups on an MA molecule to participate in acidolysis; once an amide product is formed, the second −COOH group (now attached to the amide) is sterically hindered from participating in additional decomposition reactions. Additionally, in contrast to experiments where MA was in large excess, in which the reaction mixture became a homogeneous liquid, a dry reaction mixture is formed at lower MA loadings.

We hypothesize that an initial liquefied MA adsorbs onto the PUF, and that the released repolyol is insufficient to form a liquid medium to facilitate the diffusion of MA into the remaining foam sites. MA, therefore, plays a dual role as an acidolysis reagent as well as a solvent, facilitating the transport of acid molecules within the pores of the PUF. Therefore, when a solvent-free acidolysis (no external or additional solvent, such as polyol or diols^31,32^) was performed, the initial loading of MA was critical. A PUF/MA ratio of 1:0.5 (w/w) was found to be optimal, giving a high yield of repolyol and limited amount of leftover MA and its isomer (fumaric acid) in the product mixture, making subsequent separations easier. Furthermore, this loading minimized the formation of polyol acid ester, improving the isolation yield of repolyol and reducing the use of NaOH in the subsequent hydrolysis reaction.

Prior to hydrolysis, the isolated repolyol is a viscous, brown liquid. Repolyols ideally should have low viscosity to ensure good mixing with diisocyanate to form PU. Fortunately, the viscosity of hydrolyzed repolyol falls within the accepted range of virgin polyol (Table 1). The color of the isolated repolyol deserves a comment. The color of the hydrolyzed repolyol remains dark brown. Nevertheless, the ^13^C NMR spectrum does not reveal any detectable contaminants or other byproducts. Therefore, the dark color is attributed to trace concentrations of some unknown, highly colored contaminant(s), most likely from oxidative side reactions involving aromatic amine or polyamine contaminants given the observed amine number of 1.63 for isolated repolyol. A further improvement was made to the isolation strategy using toluene/acidic water to extract the repolyol. Products isolated from this additional treatment were significantly lighter in color than the original hydrolyzed repolyol. While most literature concerning acidolysis focuses on the recovery of the polyol component of PUF, TDA obtained after hydrolysis could be a valuable coproduct from PUF acidolysis.^38,39^ Toluene diisocyanate (TDI), a precursor to PUF, is synthesized by reacting TDA with phosgene; if the TDA component of PUF can be isolated, it can be useful in the creation of new PUF materials as well.^38,40^ In our approach, a total of around 80 wt % of the input PUF can be recovered as repolyol (66–68 wt %) and TDA (12–13 wt %) for use in making new PU materials. Additionally, excess MA from acidolysis was neutralized to MA disodium salt after workup, which allows for the input MA to be recovered and recycled by reacidification.

**Figure 7:**
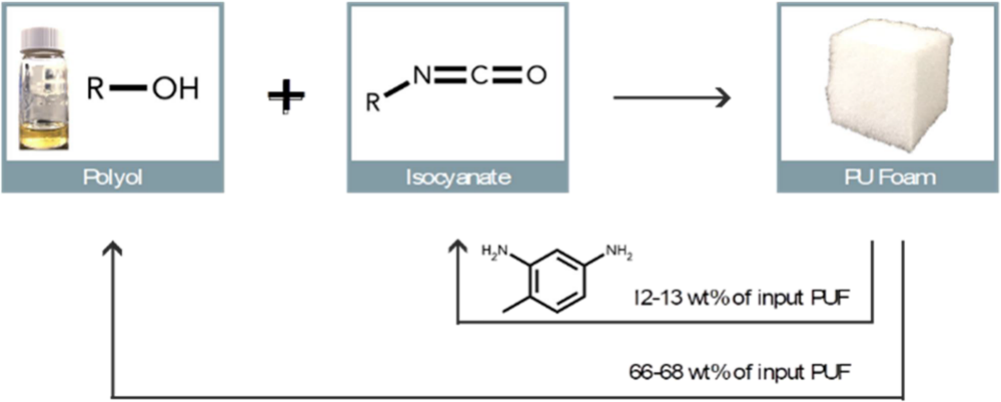
Illustration of the closed loop chemical recycling of PUF via acidolysis with MA to produce repolyol and TDA. 66–68 wt % of the input PUF is recovered as repolyol and 12–13 wt % as TDA (which is a known precursor for isocyanate).

In summary, PUF acidolysis with MA followed by hydrolysis and separations described here provides a fast and effective pathway to recycle PU through the production of repolyol and TDA under moderate temperatures (Figure 7). A 98% mass balance and up to 95% repolyol isolated yield were achieved. After hydrolysis and purification by toluene/acidic water, the key features of the isolated repolyol were identical to the virgin polyol (VORANOL 8136). The success of EOL PUF decomposition and recovery of EOL repolyol confirmed that our PUF chemical recycling method is applicable to recycling commercial waste mattresses.

## Experimental Procedures

### Resource Availability

#### Lead Contact

Further information and requests for resources should be directed to and will be fulfilled by the lead contact, Phillip Christopher (pchristopher@ucsb.edu) and Mahdi M. Abu-Omar (mabuomar@ucsb.edu).

### Materials Availability

The model PUF and VORANOL 8136 polyether polyol (the virgin polyol) was provided by The Dow Chemical Company, USA. The requests for model PUF and detailed formulation should be directed to Alan Stottlemyer (AStottlemyer@dow.com). The EOL PUF was provided by Nasim Hooshyar (NHooshyar@dow.com) from Dow in The Netherlands.

### Methods Availability

The analysis of generated repolyol was performed by The Dow Chemical Company, The Netherlands. The requests for the analytic details are provided in the Supporting Information and can be directed to Nasim Hooshyar (NHooshyar@dow.com).

### Materials and Reagents

The model PUF sample was synthesized from VORANOL 8136 polyether polyol (the virgin polyol, 71 wt % of the model PUF) and VORANATE T-80 toluene diisocyanate. The foam was prepared at an isocyanate index of 105 and a water level of 3.3 pbw with respect to 100 pbw of VORANOL 8136 polyol. See the Supporting Information for more details. Foam samples used in this study were prepared at ambient temperature (23–25 °C) in a fume hood. A high shear mixer set to a high rotational speed was used for a period of 15 s. Stannous octoate was added and immediately mixed for an additional 15 s. Finally, the isocyanate sample was added to the mixture immediately, followed by mixing for 3 s. The resulting mixture was poured into a plastic film-lined wooden box of dimensions 38 cm × 38 cm × 24 cm (*L* × *W* × *H*). Once foaming was complete, the resulting foam sample was allowed to cure overnight in a fume hood. Accordingly, the urethane bond density (carbamate density) within the model PUF was 0.71 mmol/g, while the urea bond density was 1.43 mmol/g (Table S2). The EOL PUF was collected from commercial waste in Europe. The EOL-PUF was received as a mixture of different colored foam with an average particle size of around 1–2 cm^3^. The EOL PUF was a random mixture of mattress waste, of which the type and content of polyol used in the EOL PUF sample were unknown. The waste foam was not analyzed spectroscopically but was sorted according to grade/type. The foams were presorted and viscoelastic ones removed as it is easy to distinguish from conventional foam.

Maleic acid (≥99%) was purchased from Sigma-Aldrich. Calcium oxide (CaO, reagent grade) was purchased from Sigma-Aldrich. Sodium hydroxide (NaOH) was purchased from Spectrum Chemical Mfg. Corp. Ethyl acetate (EtOAc, ACS reagent ≥99.5%) was purchased from Sigma-Aldrich. Toluene (ACS reagent) was purchased from Fisher Chemical. Chromium(III) acetylacetonate (Cr(acac)_3_ 99.99%) was purchased from Sigma-Aldrich. Hexadeuterated dimethyl sulfoxide (DMSO-d_6_, 99.9%) was purchased from Cambridge Isotope Laboratories Inc. The high purity nitrogen gas (N_2_) cylinder was purchased from Praxair Technology Inc. DI water was obtained from the Milli-Q EQ 7000 ultrapure water purification system. Purchased chemicals were used as received.

### Grinding Pretreatment

Before the acidolysis reaction, both model PUF and EOL-PUF chunks were ground into smaller particles. The chunks of flexible foam were first flash-frozen in liquid nitrogen to increase their brittleness and transferred subsequently to a grinder equipped with cross blades. After grinding, the particle size of model PUF was between 500 and 2000 μm, while the EOL-PUF particles were around 155–750 μm.

### PUF Acidolysis Reaction Setup

The acidolysis reaction was carried out in a glass reaction system. In this setup, a 250 mL round-bottom flask was connected to a coldfinger that was connected through Tygon tubing to either a gas evolution buret or a filtration flask with saturated calcium hydroxide (Ca(OH)_2_) solution. The liquid products were collected in a round-bottom flask while evolved carbon dioxide (CO_2_) gas captured by the gas evolution buret or isolated as calcium carbonate (CaCO_3_) in the filtration flask. Saturated Ca(OH)_2_ was prepared by mixing excess CaO with DI water at room temperature. For each reaction, ground PUF was premixed with maleic acid in the round-bottom flask, and the reaction mixture was purged with N_2_. Magnetic stirring at 250 rpm and heat through a regulated oil bath were applied. After the reaction, the product mixture was cooled to room temperature, washed with EtOAc, and vacuum-filtered through a Buchner funnel equipped with filter paper. The solid residue (if any) was collected and dried under vacuum. The liquid filtrate was transferred to a 50 mL centrifuge tube for product separation.

### Product Separation

In each 50 mL centrifuge tube, the liquid products were dissolved in 30 mL of EtOAc solution and mixed with 20 mL of NaOH aqueous solution (50 wt %/wt, aq). Prior to centrifugation, the two liquid phases were well mixed by a mechanical shaker for 5 min to enhance product extraction. The centrifugation was performed at 7000 rpm for 10 min. Next, the liquid in the tube separated into three phases; the top EtOAc phase contained amides and some repolyol products, the middle phase was repolyol with some water-EtOAc mixture, and the bottom aqueous phase contained leftover excess maleic acid, amides, and other byproducts. The top phase could be further purified by repeating the centrifugation process with a NaOH solution to remove amides from the EtOAc phase. After purification, the repolyol from the top EtOAc phase and the middle phase were combined. The repolyol product was dried by rotary evaporation to remove residual EtOAc and water. The byproducts in the aqueous phase were also collected by removing water through a rotovap. The repolyol and product in the aqueous phase were analyzed.

### Hydrolysis and Further Purification Treatment

Two hydrolysis treatments were performed. One hydrolysis approach was composed of a three-step process: (1) The repolyol middle phase from centrifugation was separated; (2) the collected repolyol was subjected to liquid–liquid extraction in EtOAc followed by solvent removal and drying; (3) the dried repolyol product was transferred into a round-bottom flask charged with 25 mL of NaOH aqueous solution (NaOH was added by 1:1 molar ratio with respect to the total −COOH from MA used in the PUF acidolysis reaction). The reaction mixture was stirred and heated in a 150 °C oil bath to maintain reflux. Typical hydrolysis for repolyol obtained from model PUF required 25–30 min reaction time, while 120 min was necessary for complete hydrolysis of repolyol obtained from EOL PUF.

An alternative hydrolysis involved two steps and was performed with the crude reaction mixture from acidolysis bypassing centrifugation. (1) At the end of PUF acidolysis ca. 15 min of reaction, 25 mL of precalculated NaOH solution (1:1 molar ratio with respect to the total −COOH from MA used in the PUF acidolysis reaction) was added, and the oil bath temperature was lowered from 175 to 150 °C maintaining reflux for 25–30 min (model PUF) or 120 min (EOL PUF). (2) After hydrolysis workup, the reaction mixture was cooled to room temperature, dissolved in 50 mL of DI water and 100 mL of toluene, and transferred to a 250 mL separatory flask. The mixture was placed in a separatory flask overnight for phase separation. The bottom aqueous layer was collected from the bottom of the separatory flask, while the top toluene layer was poured from the top of the flask. The aqueous layer had TDA products, while the repolyol product was isolated in the toluene layer. By comparison, the aqueous layer from the three-step hydrolysis approach could be discarded because it contained no TDA product; however, the aqueous layer from this two-step hydrolysis required further liquid–liquid extraction with EtOAc and centrifugation (7000 rpm for 20 min) to remove TDA products from the aqueous phase into EtOAc. The toluene layer was also transferred to a plastic centrifuge tube. For each 30 mL of toluene solution, 25 mL of water and 1 mL of HCl (36%) were added to the tube and mixed well. By adding HCl to the mixture, the pH was adjusted to slightly acidic between pH 6 and 5. This facilitates the solubility of impurities into the aqueous phase while leaving the repolyol in the toluene phase. The mixture was centrifuged for 45 min at 7500 rpm. After that, the top orange toluene layer was carefully collected from the centrifuge tube into a round-bottom flask. Hydrolyzed repolyol was finally collected after the removal of toluene using rotovap. The TDA product was confirmed by GC-MS and ^1^H–^13^C HSQC NMR, while the repolyol was analyzed by ^13^C NMR. The isolated neat TDA and repolyol were also quantified by weight.

### Mass Balance

The mass balance of PUF acidolysis was obtained by comparing the total weight of gas, liquid, and solid products versus the starting weight of PUF and maleic acid. The overall yield of repolyol from PUF acidolysis after hydrolysis and purification was calculated based on the obtained neat repolyol versus the polyol content within the input PUF.

### ^13^C and ^1^H–^15^N HSQC Nuclear Magnetic Resonance Spectroscopy

The products from PUF acidolysis were identified by ^13^C NMR with a Bruker Avance NEO 500 MHz spectrometer, which was equipped with a 5 mm X-nuclei optimized double resonance cryoprobe. For each measurement, 100–150 mg of the sample was dissolved in 600 μL of DMSO-d6 and packed in a 5 mm glass tube. For quantitative 13C NMR analysis, 100 μL of 25 mM Cr(acac)_3_ was added to 150 mg of the sample with 600 μL of DMSO-d_6_ and 100 mg of methanol (internal standard) in the 5 mm NMR glass tube. The chemical shifts of the polyol (CAS# and name: 9082–00–2, glycerol, propylene oxide, ethylene oxide polymer) were assigned accordingly for a glycerol, propylene oxide, and ethylene oxide polymer. The key assignments, such as the ^13^C shifts (δ^13^C) of carbon with the OH ending group on polyol, were between 65 and 66.7 ppm (major) and 60.3–62.1 ppm (minor). The δ^13^C values between 67.5 and 79.5 ppm are the carbon backbones of the polyol. The δ^13^C between 16.5 and 19.5 ppm are the methyl branches on the polyol molecule. For instance, the quantitative ^13^C NMR of polyol products was determined by integrating the δ^13^C between 74.2 and 76.0 ppm compared to the integral of 100 mg internal standard between δ^13^C 48.9–49.1 ppm (number of resonating carbon set to 1): *m*_polyol_/*m*_methano_ = *A*_polyol_/*A*_methanol_ where the *m*_polyol_ and *m*_methanol_ are the mass of analyte, *A*_polyol_ and *A*_methanol_ are the integral area. *A*_methanol_ was set to 1 as reference, while *m*_methanol_ was known (100 mg). Thus, the mass of polyol could be calculated as *m*_polyol_ = 100 mg × *A*_polyol_. The amide carbons were observed at δ^13^C 110–140 and 160–174 ppm. The MA signals were assigned by comparison to a pure MA standard, which had shifts at δ^13^C 130.5 and 167.2 ppm. The ^1^H–^15^N HSQC NMR spectrum was acquired under the same sample preparation with the same NMR spectrometer. The assignments of the amine region on HSQC NMR were done according to the literature.^41^

### Thermogravimetric Analysis

TGA was carried out by a Discovery 5500 Thermogravimetric Analyzer. For each measurement, 5–10 mg of the solid sample was loaded into an Al_2_O_3_ ceramic crucible. The crucible was then placed on a high temperature platinum sample pan, which was calibrated prior to each measurement. To analyze the PUF samples, the crucible was loaded by autosampler to the TGA chamber, which was well insulated and protected under 25 mL/min N_2_ flow. The TGA chamber was first heated to 50 °C for 5 min, and the moisture content within the PUF sample was determined by the weight loss during this period. Then, the chamber was heated to 550 °C at a ramping rate of 20 °C/min and held for 10 min. The weight change between 220 and 320 °C was assigned to PUF hard segments (polymeric linkages composed of diisocyanate carbon backbones and its short chain linkages), while the weight change between 320 and 450 °C was assigned to PUF soft segments (polymeric linkages composed of long chain polyol). The chamber was then heated to 650 °C at a ramping rate of 30 °C/min under a 25 mL/min flow rate of air. The weight percentage of char (TGA residue left in the crucible) was acquired after holding the chamber at 650 °C for 5 min.

### Scanning Electron Microscopy

SEM images were acquired on an FEI Nova Nano 650 FEG SEM microscope, which is equipped with a high stability Schottky field emission gun and large specimen chamber. The PUF sample was loaded onto double-sided copper foil tape with conductive adhesive to attach the sample onto a SEM aluminum specimen stub. Prior to the imaging, the PUF sample was first coated with gold (Au) by applying 10 mA plasma over 90 s under 90 mTorr Ar. Then, the Au-coated PUF sample was transferred to a SEM specimen chamber. The chamber was evacuated to 8 × 10^–5^ mbar pressure during the measurement. The PUF images were observed through an Everhart-Thornley detector (ETD) with the beam voltage between 3 and 5 keV under high resolution secondary election mode (SE mode).

### Gel Permeation Chromatography

*M*_n_, *M*_w_, M_*z*_, and polydispersity (*Đ* = *M*_w_/*M*_n_) of virgin polyol and repolyol samples were determined by using gel permeation chromatography (GPC). The GPC analyses were performed using a Waters Acquity APC system with three Acquity APC XT columns and an Acquity UPLC refractive index detector. The eluent used was THF and was pumped at a rate of 0.9 mL/min. For each sample, approximately 12 mg of polyol/repolyol was dispersed in 2 mL of THF and was left standing for at least one hour to allow for the full solvation of the sample. The solutions were then filtered through a 0.45 μm PTFE membrane (Millipore) before injection. The injection volume to the columns was 25 μL, and the column was held at 35 °C. The GPC columns were calibrated using polystyrene standards.

### Gas Chromatography–Mass Spectroscopy

The TDA product was determined by GC-MS, and the spectra of MS fragmentations were compared to the NIST (National Institute of Standards and Technology) database to identify TDA. Shimadzu GC-2010 equipped with an Agilent DB-1 capillary column (dimethylpolysiloxane, 30 m × 0.25 mm × 0.25 μm) coupled with a QP2010 mass spectrometer was used. The recovered TDA sample from PUF acidolysis was dissolved in chloroform and packed in a 2 mL GC vial. Prior to injection, the GC injector and detector were preset to 250 °C with a 10 mL/min helium flow rate. The column oven was equilibrated at 40 °C for 3 min. After sample injection, the GC column was heated to 250 °C at a ramping rate of 25 °C/min and held for 10 min. The determined GC-MS fragmentation patterns of the TDA product gave a 97% match to the NIST MS database of toluene-2,4-diamine (or 4-methylbenzene-1,3-diamine).

## Supporting Information Available

NMR, FT-IR, GPC, TGA, SEM images, MS, and DFT calculations (PDF)
