# Supplementary material for: Polyurethane Foam Chemical Recycling: Fast Acidolysis with Maleic Acid and Full Recovery of Polyol
Source: ACS Sustain Chem Eng. 2024 Mar 7;12(11):4435–43. doi: 10.1021/acssuschemeng.3c07040 (PMC10952008; doi:10.1021/acssuschemeng.3c07040)
Supplement: Supplementary file 1 — sc3c07040_si_001.pdf [file sc3c07040_si_001.pdf]

SUPPORTING INFORMATION

FOR

Polyurethane Foam Chemical Recycling: Fast Acidolysis with Maleic Acid and Full Recovery of Polyol

Baoyuan Liu,[1†] Zach Westman,[2] Kelsey Richardson,[2] Dingyuan Lim,[1] Alan Stottlemyer,[3] Thomas Farmer,[3] Paul Gillis,[3] Nasim Hooshyar,[4] Vojtech Vlcek,[1] Phillip Christopher,[2,5*] and Mahdi M. Abu-Omar[1,2,5**]

[1]Department of Chemistry and Biochemistry, University of California, Santa Barbara, CA 93106, United States

[2]Department of Chemical Engineering, University of California, Santa Barbara, CA 93106, United States

[3]The Dow Chemical Company, Midland, MI 48640, United States

[4] The Dow Chemical Company, Herbert H Dowweg 5, 4542 NH Hoek, The Netherlands

[5]Lead contact

*Correspondence: pchristopher@ucsb.edu

**Correspondence: mabuomar@ucsb.edu

Table of contents:

Page S2: **Table S1** Properties of common organic dicarboxylic acids for PUF acidolysis

Table S2 Model PUF components/preparation

Page S3: **Table S3** Calculations of molar content for urethane and urea bonds in model PUF

Page S4: **Figure S1** SEM images of PUF samples

Page S5: **Figure S2** FT-IR spectra of PUF samples

Page S5: **Figure S3** TGA analysis of model and EOL PUF samples

Page S6: **Figure S4** ATR FT-IR spectra of maleic acid, fumaric acid, and acidolysis solid residue

Page S7: **Figure S5** Reaction setup and mass balance of PUF acidolysis at PUF/MA 1:2 for 3 hr

Page S8: **Figure S6** Gas evolution burette acidolysis setup

Page S9: **Figure S7** APC GPC THF spectra of polyol samples

Page S10: **Figure S8** Mass Spectrometry analysis of TDA products from model PUF

Page S11: **Figure S9** 2D ^{1}H-^{15}N HSQC NMR of TDA products from model PUF

Page S12 – S14: DFT calculations

S12: **Figure S10** Example of PUF monomeric unit

Figure S11 Simplification of PUF monomeric unit

S13: **Figure S12** Simplified PUF acidolysis reaction scheme

Figure S13 Free energy of PUF acidolysis calculated with B3LYP and PBE functionals

S14: **Figure S14** Free energy of PUF acidolysis calculated with PBE functional

Figure S15 Free energy of PUF acidolysis reaction

Page S15: **Figure S16** Comparison of repolyol obtained from different purification methods

Page S16: References

Table S1: Properties of common organic dicarboxylic acids for PUF acidolysis

	m.p. (℃)	pKa_1	pKa_2
Maleic acid	136	1.90	6.07
Succinic acid	184	4.20	5.60
Phthalic acid	210	2.89	5.50
Adipic acid	152	4.43	5.41

Table S2. Model PUF components/preparation.

**Component**	**Type**	**Parts by Weight**
VORANOL™ 8136	polyether polyol	100
VORASURF™ DC 5987	silicone surfactant	0.8
Dabco 33LV	amine catalyst	0.08
Niax A-1	amine catalyst	0.03
Dabco T-9	tin catalyst	0.18
Water	water	3.3
VORANATE™ T-80	toluene diisocyanate (TDI)	42.7 (Iso Index = 105)

Table S3. Formulation of model PUF and examples of calculation

Component	Mass input (g)	M_w (g/mol)	Molar input (mole)	Functionality	Equivalent molar functionality (mole)	Mass %
VORANOL™ 8136	100	3090	0.032	3	0.097	72.7*
VORANATE™ T-80	42.7	174.2	0.245	2	0.49	27.3
Water	3.522	18	0.195	2	0.39	
CO_2 (mass loss of PUF synthesis)	-8.65	44	-0.197			
**Resulting PUF (g)**	137.57					
	**Total (mole)**	**Molar density (mmol/g PUF)**		**Equivalent TDI functionality (mole)**	**Equivalent polyol functionality (mole)**	
**Resulting urethane**	0.097	0.71		0.097	0.097	
**Resulting urea**	0.197	1.43		0.393	N/A	

* Mass% of polyol in model PUF used for calculations was 71% due to presence of leftover catalyst.

Example of calculations:

Molar input: $\frac{Mass\ input}{M_w}$

Equivalent molar functionality: (Molar input) × Functionality

Polyol was added as limiting reagent for PUF synthesis, thus the mass% calculated for polyol represents the urethane component in resulting PUF. Similarly, the TDI and water were reacted to form urea in the resulting PUF and thus their mass% was added together. CO_2 was produced and released during the urea formation. Therefore, the mass of CO_2 (negative mass input) was subtracted from the overall mass.

Resulting PUF: sum of Mass input of all components.

Mass% of VORANOL™ 8136 (polyol): $\frac{Mass\ input\ of\ polyol}{Resulting\ PUF}$

Mass% of VORANATE™ T-80 TDI and Water:

$$\frac{Mass\ input\ of\ TDI + Mass\ input\ of\ water + Mass\ input\ of\ CO_2}{Resulting\ PUF}$$

According to the formulation of model PUF, polyol and water are added as limiting reagent. Therefore, the moles of input polyol represent the moles of resulting urethane while the moles of water equal the moles of urea.

Molar density: $\frac{millimoles\ of\ Resulting\ urethane\ (or\ urea)}{Resulting\ PUF\ (g)}$

For example, 3 g PUF was used for acidolysis reaction. After the acidolysis reaction, 2 g of repolyol and 0.4 g of TDA were collected after hydrolysis and purification. The yield of repolyol and TDA was calculated as (by mass):

Yield of repolyol: $\frac{2\ g}{3\ g} \times 100\% = 66.7\ wt\%$

Yield of TDA: $\frac{0.4\ g}{3\ g} \times 100\% = 13.3\ wt\%$

Total recovery of repolyol and TDA from starting PUF: 66.7 wt% + 13.3 wt% = 80 wt%.

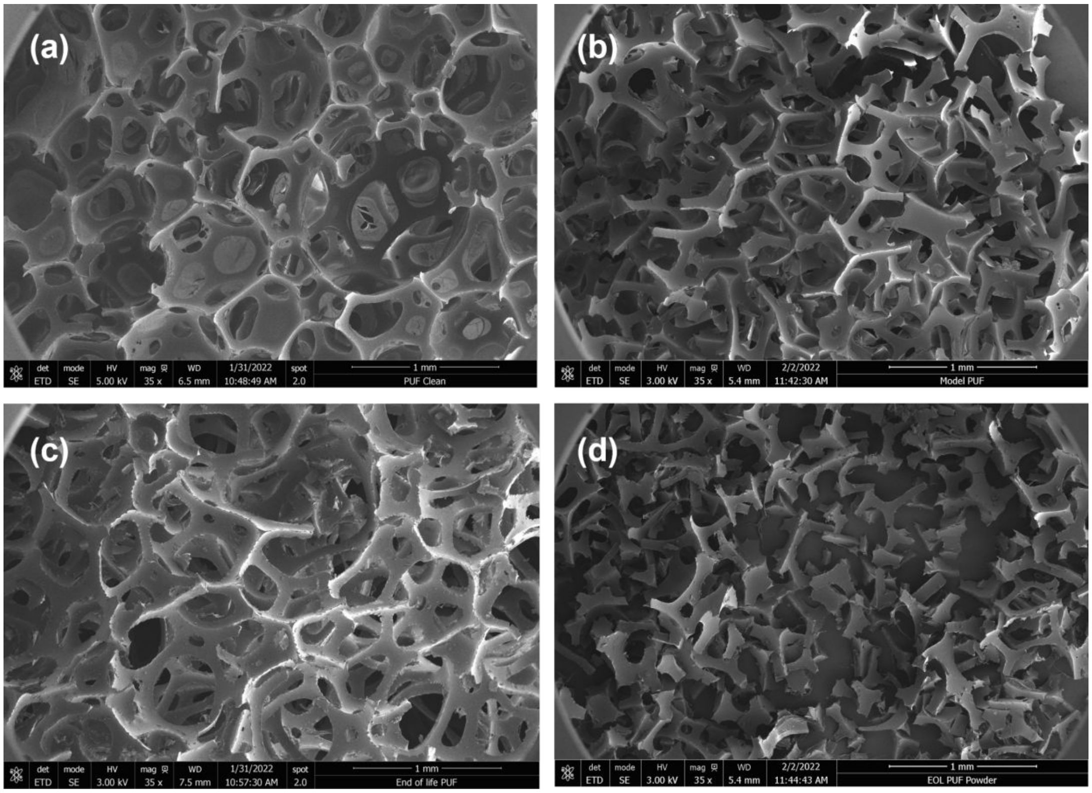

Figure S1. SEM images of (a) Intact model PUF; (b) shredded model PUF; (c) intact EOL PUF; (d) shredded EOL PUF.

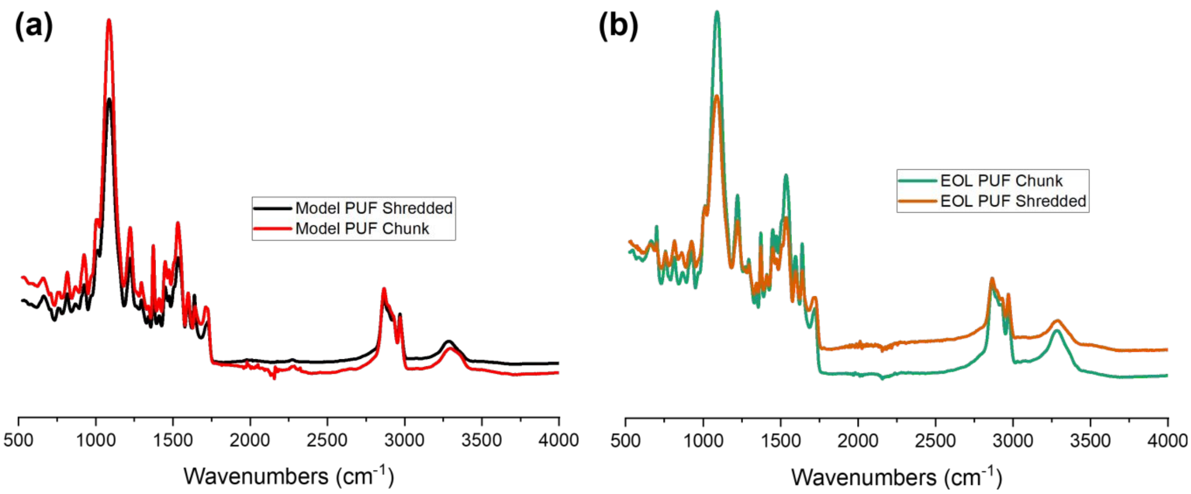

Figure S2: FT-IR spectra of (a) comparison between intact model PUF vs shredded model PUF; (b) comparison between intact EOL PUF vs. shredded EOL PUF.

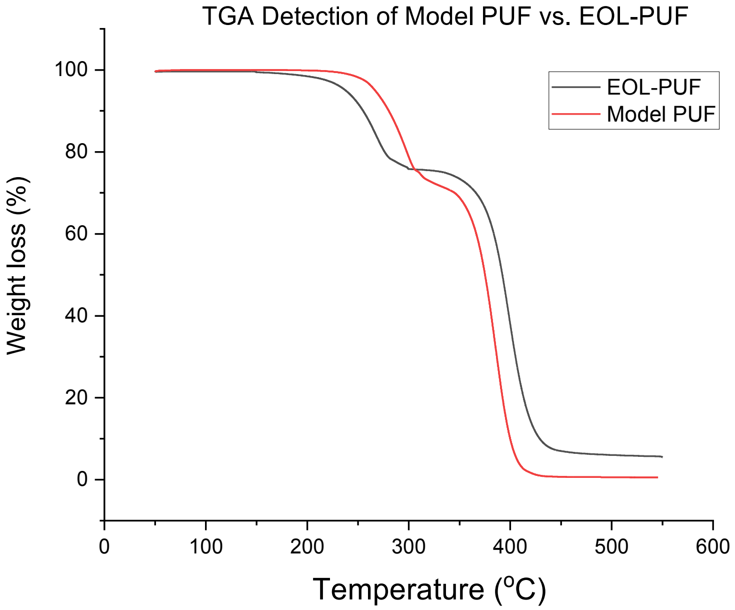

Figure S3: TGA analysis of model and EOL PUF. The weight loss (%) was determined based on temperature. The TGA analysis was carried out under N2. The weight change between 220 – 320 °C was assigned to hard segment decomposition while the weight change between 320 – 450 °C was assigned to soft segment decomposition. The assignments of hard and soft segment weight/content were based on the known composition of the model PUF formulation provided by Dow.

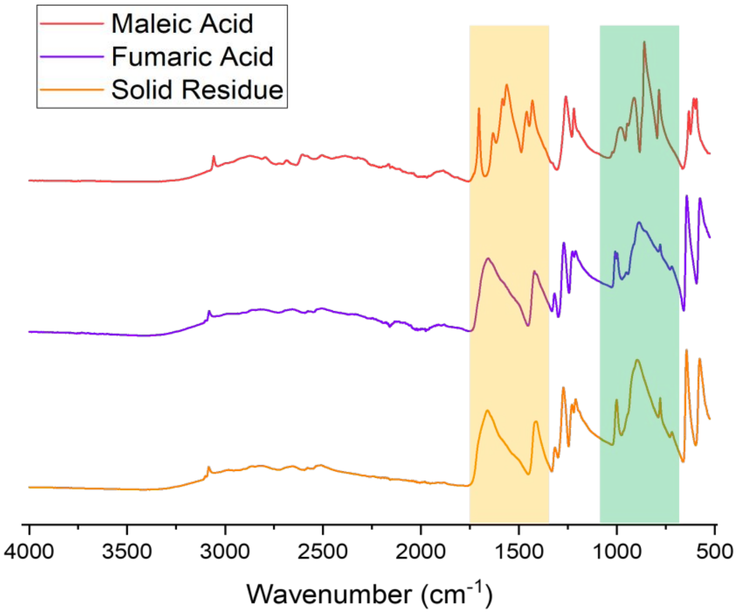

Figure S4. ATR FT-IR spectra of maleic acid, fumaric acid, and solid residue obtained from 3 h acidolysis reaction at 175°C.

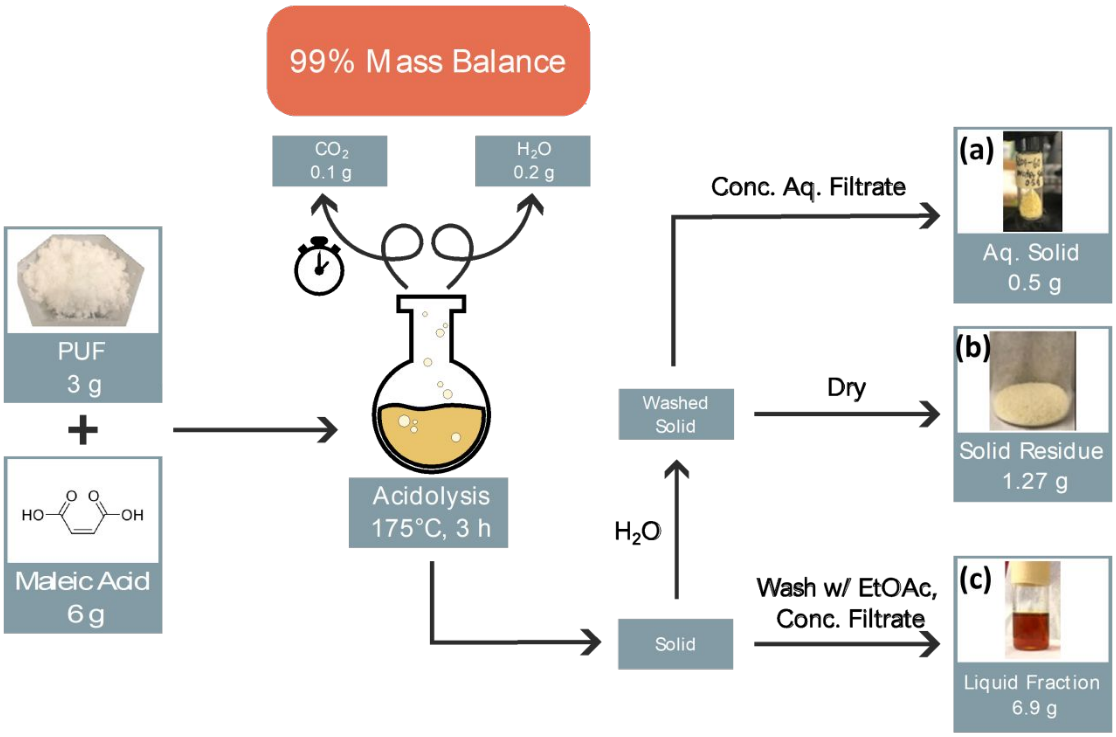

Figure S5. Reaction workup and mass balance of a typical acidolysis of model PUF with MA at PUF/MA 1:2 ratio. Acidolysis conditions: 175 °C for 3 h. The cooled post reaction mixture was washed with water and EtOAc followed by liquid-liquid phase separation via centrifuge. The fraction (a) was the dried solute obtained from aqueous phase; the fraction (b) was a solid component observed and dried after reaction that was insoluble in both aqueous and organic phase; the fraction (c) was the remaining component obtained from organic phase after removal of EtOAc. Excess water generated from the 3-hour reaction (as compared to Figure 2) is attributed to dehydration of MA to form maleic anhydride. Mass balance calculation:

$$\frac{Total\ mass\ collected\ of\ (a)Aq.Solid + (b)Solid\ residue + (c)Liquid\ fraction + CO_2 + H_2O}{Total\ mass\ input\ of\ PUF + Maleic\ acid} \times 100\% =$$

$$\frac{0.5\ g\ +\ 1.27\ g + \ 6.9\ g + 0.1\ g + 0.2\ g}{3\ g + 6\ g} \times 100\% = 99.67\%$$

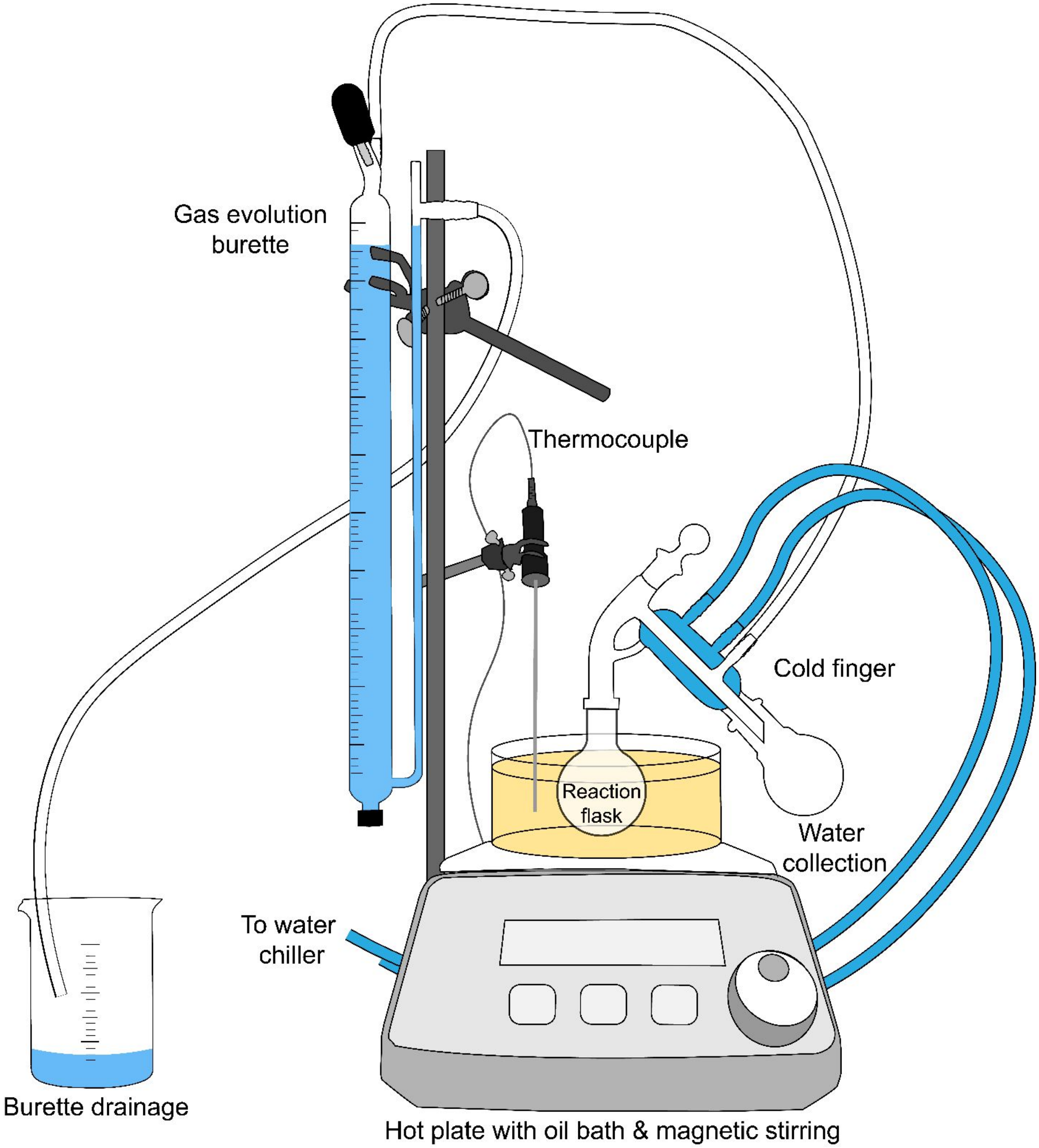

Figure S6. Gas evolution burette setup used to monitor acidolysis. Volume in burette was recorded at beginning of reaction and at each time point selected, and the volume of gas produced was calculated as $V_g(t) = V_l(0) – V_l(t)$, where V_g and V_l are volumes of gas produced and liquid in the burette, respectively. Time = 0 was chosen to be the time at which the reaction flask reached 175 °C (i.e., the time at which gas evolution from thermal expansion was observed to cease).

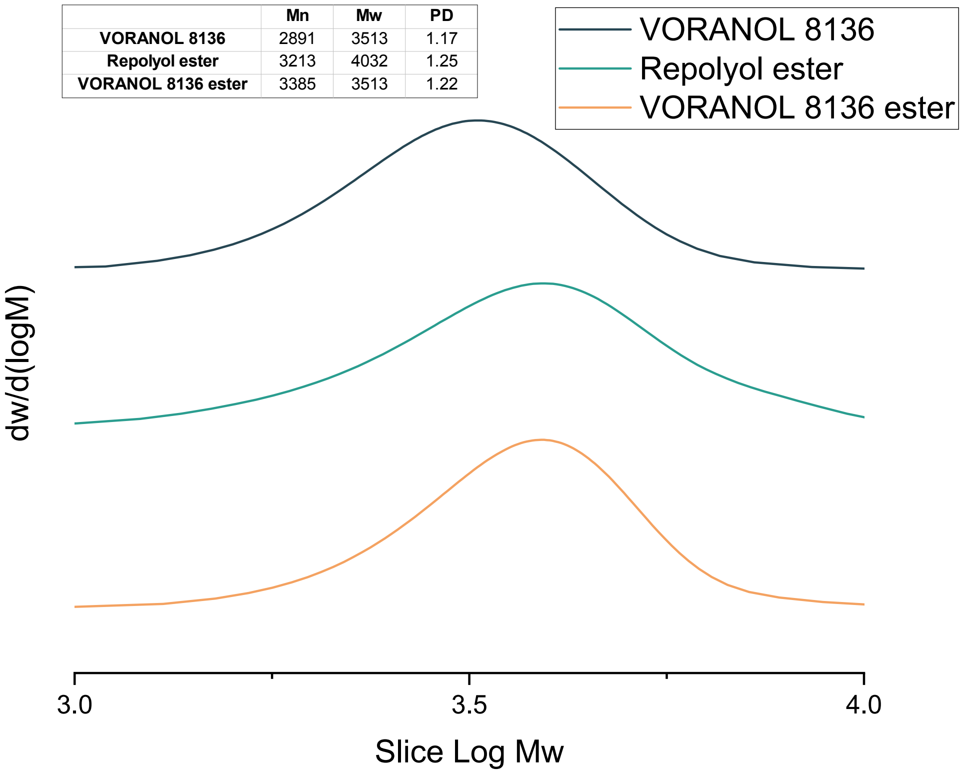

	Mn	Mw	PD
**VORANOL 8136**	2891	3513	1.17
**Repolyol ester**	3213	4032	1.25
**VORANOL 8136 ester**	3385	3513	1.22

Figure S7. APC GPC THF of a repolyol ester from acidolysis with maleic acid at 175 ℃, along with two references: VORANOL™ 8136 polyol and its ester with maleic acid, produced from heating VORANOL™ 8136 and maleic acid at 175 ℃ for 3 h.

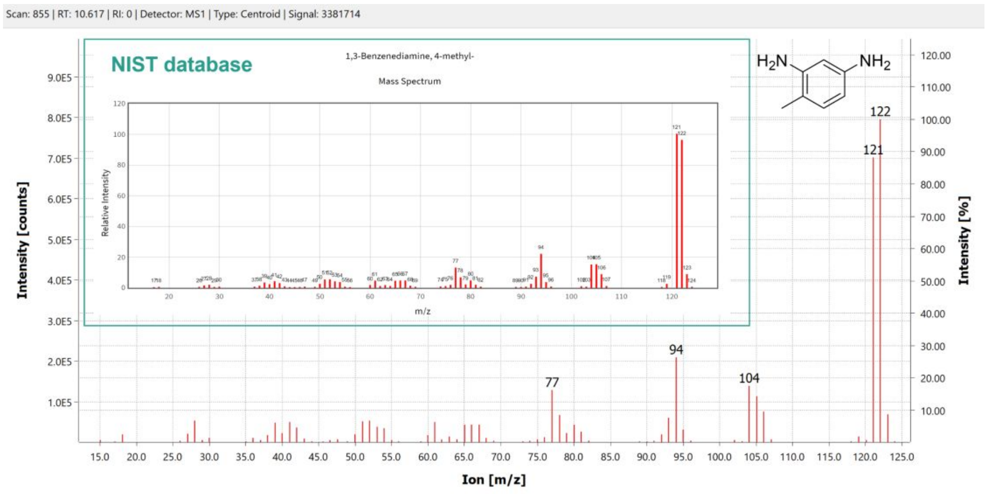

Figure S8. Mass spectrum (MS) obtained from GC-MS of isolated TDA from model PUF acidolysis with MA after hydrolysis and workup. Inset: NIST MS of authentic TDA standard. 97% match to isolated TDA product from PUF acidolysis.

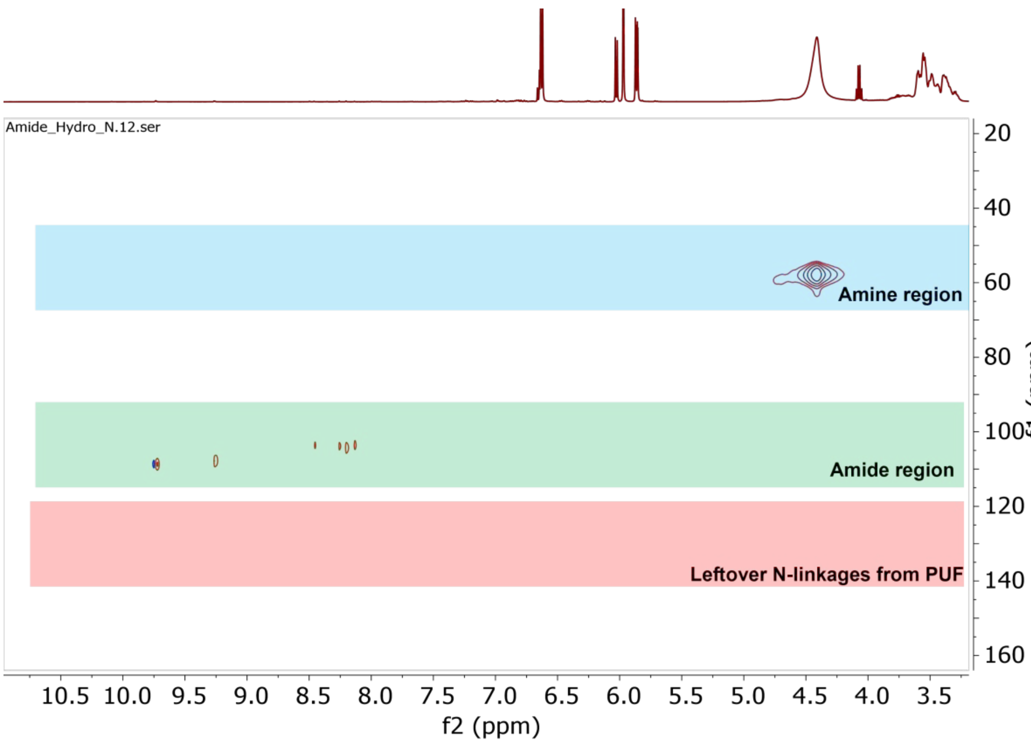

Figure S9. 2D ^{1}H-^{15}N HSQC NMR of isolated TDA from model PUF acidolysis with MA after hydrolysis and workup. The TDA was observed in the Amine region (blue colored); trace amount of amide product as TDI-derivative was observed in Amide region (green colored); no other N-contained product was observed in region with red color indicates the full decomposition of model PUF through acidolysis with MA.

DFT Calculations

Geometry optimizations using the cc-pVTZ correlation consistent basis set and the Perdew,[1, 2] Burke, and Ernzhof (PBE) exchange-correlation functional were implemented in Gaussian 16.[3] The reaction enthalpy, entropy, and free energies were defined as the enthalpy, entropy, and free energy differences between the final and initial states. No solvation model was used, so all calculations were carried out in vacuum.

Simplified Reaction Scheme Determination

Figure S-9 shows a monomer of PUF. The complexity of the PUF monomer is computationally prohibitive for a high number of calculations. To lower the computational burden of the calculations, we simplified the reaction system as shown in Figure S10.

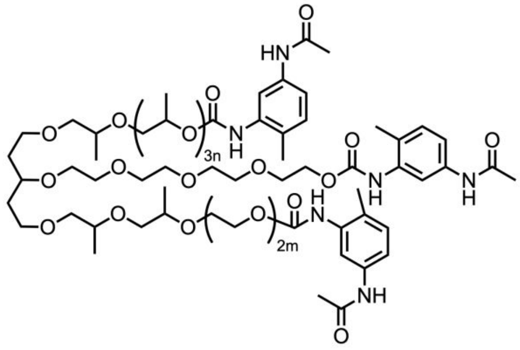

Figure S10. Example of a PUF monomeric unit

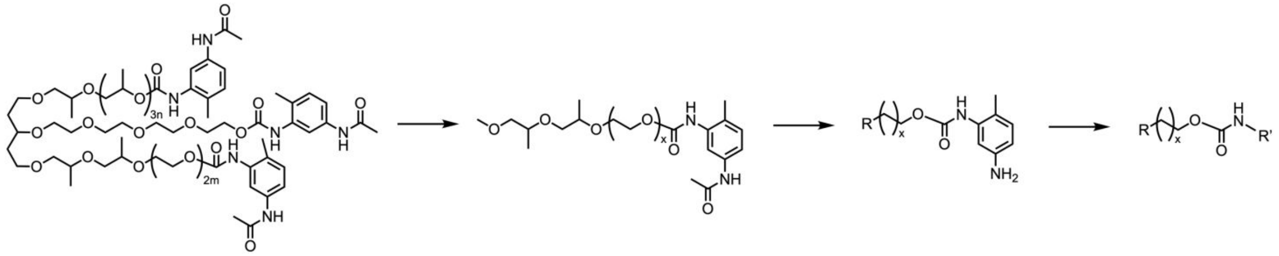

Figure S11. Simplification of PUF monomeric unit

We hypothesize that the results of our proposed calculations with this simplified system will scale to understand the chemistry at the polymer scale. To do this, we tested different chain lengths, represented by ‘x’ in Figure S10, to understand how increasing the size of the carbon chain in PU affects the thermodynamics of acidolysis. We determined that the chemistry of acidolysis is very local, indicated by the non-changing free energy with increasing ‘x’. As a result, we used ‘x’ = 1 for future computations. The resulting simplified PUF acidolysis reaction scheme is shown in Figure S11.

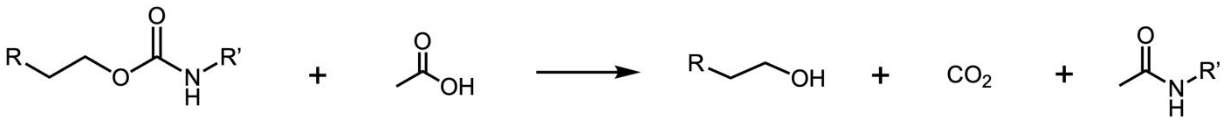

Figure S12. Simplified PUF acidolysis reaction scheme

Basis Set and Functional Selection

B3LYP and PBE are two common exchange-correlation functionals for organic systems. The thermodynamic results, overall free energy of PUF acidolysis at different temperatures in the range 100-180 °C, are shown in Figure S13 for two functionals. B3LYP and PBE yield the same free energy of acidolysis within 15%. PBE, however, requires less computational time and was thus selected as the functional for future computations.

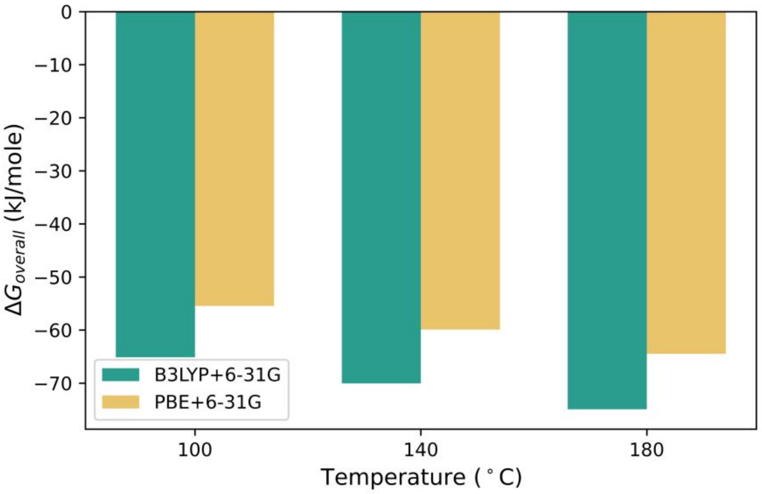

Figure S13. Free energy of PUF acidolysis calculated with B3LYP and PBE functionals. Reaction from Fig. S12. R=R'=methyl.

Next, we tested three different basis sets for the calculation of thermodynamics of PUF acidolysis: 6-31G, cc-pVTZ, and cc-pVQZ. The results of these calculations, done with PBE functional, are shown in Figure S-13.[1, 4, 5] From this, we selected cc-pVTZ as it yielded the same results as cc-pVQZ, a larger and thus more accurate basis set, with smaller computational time.

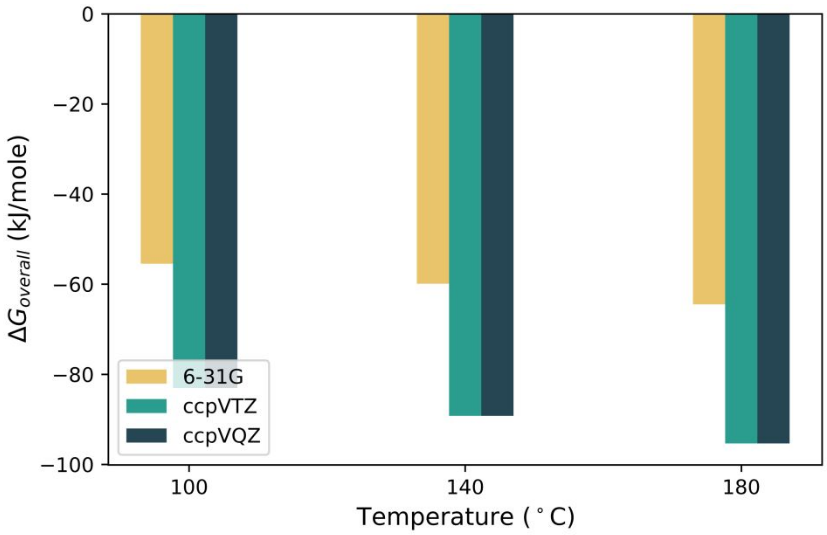

Figure S14. Free energy of PUF acidolysis calculated with PBE functional and 6-31G(d), cc-pVTZ, and cc-pVQZ basis sets. Reaction from Fig. S11. R=R'=methyl.

Free Energy of Acidolysis

With PBE functional, cc-pVTZ basis set, and selected representative reaction (Figure S11), we calculated the free energy of acidolysis at the pertinent reaction temperatures (Figure S14). From this, we determined that the acidolysis of PUF is thermodynamically viable, which is consistent with the observed experimental results.

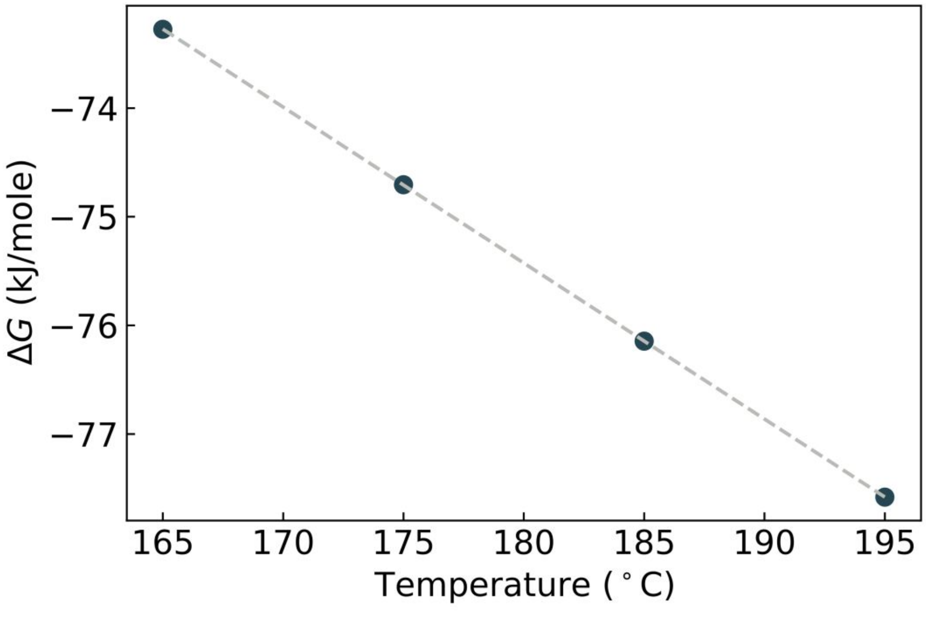

Figure S15. Free energy of PUF acidolysis reaction in Fig. S11, R=R'=methyl, cc-pVTZ basis set, PBE functional, no solvation. Line added to guide the eye.

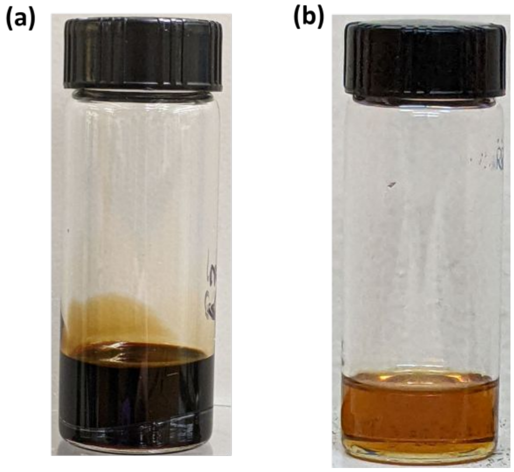

Figure S16. Comparison between original hydrolyzed repolyol vs. the toluene/acid purification treated repolyol from model PUF. (a) shows the original hydrolyzed repolyol obtained from NaOH hydrolysis and isolated by EtOAc; (b) shows the hydrolyzed repolyol obtained from NaOH hydrolysis and isolated and purified by toluene/acid treatment.

References

1. Kendall, R. A.; Dunning, T. H.; Harrison, R. J., Electron affinities of the first-row atoms revisited. Systematic basis sets and wave functions. *The Journal of Chemical Physics* **1992,** *96* (9), 6796-6806.
2. Perdew, J. P.; Burke, K.; Ernzerhof, M., Generalized Gradient Approximation Made Simple. *Physical Review Letters* **1996,** *77* (18), 3865-3868.
3. Frisch, M. J.; Trucks, G. W.; Schlegel, H. B.; Scuseria, G. E.; Robb, M. A.; Cheeseman, J. R.; Scalmani, G.; Barone, V.; Petersson, G. A.; Nakatsuji, H.; Li, X.; Caricato, M.; Marenich, A. V.; Bloino, J.; Janesko, B. G.; Gomperts, R.; Mennucci, B.; Hratchian, H. P.; Ortiz, J. V.; Izmaylov, A. F.; Sonnenberg, J. L.; Williams; Ding, F.; Lipparini, F.; Egidi, F.; Goings, J.; Peng, B.; Petrone, A.; Henderson, T.; Ranasinghe, D.; Zakrzewski, V. G.; Gao, J.; Rega, N.; Zheng, G.; Liang, W.; Hada, M.; Ehara, M.; Toyota, K.; Fukuda, R.; Hasegawa, J.; Ishida, M.; Nakajima, T.; Honda, Y.; Kitao, O.; Nakai, H.; Vreven, T.; Throssell, K.; Montgomery Jr., J. A.; Peralta, J. E.; Ogliaro, F.; Bearpark, M. J.; Heyd, J. J.; Brothers, E. N.; Kudin, K. N.; Staroverov, V. N.; Keith, T. A.; Kobayashi, R.; Normand, J.; Raghavachari, K.; Rendell, A. P.; Burant, J. C.; Iyengar, S. S.; Tomasi, J.; Cossi, M.; Millam, J. M.; Klene, M.; Adamo, C.; Cammi, R.; Ochterski, J. W.; Martin, R. L.; Morokuma, K.; Farkas, O.; Foresman, J. B.; Fox, D. J. *Gaussian 16 Rev. C.01*, Wallingford, CT, 2016.
4. Woon, D. E.; Dunning, T. H., Gaussian basis sets for use in correlated molecular calculations. III. The atoms aluminum through argon. *The Journal of Chemical Physics* **1993,** *98* (2), 1358-1371.
5. Ditchfield, R.; Hehre, W. J.; Pople, J. A., Self-Consistent Molecular-Orbital Methods. IX. An Extended Gaussian-Type Basis for Molecular-Orbital Studies of Organic Molecules. *The Journal of Chemical Physics* **1971,** *54* (2), 724-728.
